# Huntington’s disease phenocopy syndromes revisited: a clinical comparison and next-generation sequencing exploration

**DOI:** 10.1136/jnnp-2024-333602

**Published:** 2024-10-23

**Authors:** Carolin Anna Maria Koriath, Fernando Guntoro, Penelope Norsworthy, Egor Dolzhenko, Michael Eberle, Davina J Hensman Moss, Michael Flower, Holger Hummerich, Anne Elizabeth Rosser, Sarah J Tabrizi, Simon Mead, Edward J Wild

**Affiliations:** 1 LMU University Hospital, Department of Psychiatry and Psychotherapie, Ludwig Maximilian University of Munich, Munchen, Bayern, Germany; 2 MRC Prion Unit at the UCL Institute of Prion Disease, London, UK; 3 Illumina Inc, San Diego, California, USA; 4 St George’s University of London, London, UK; 5 Department of Neurodegenerative Disease, UCL Queen Square Institute of Neurology, London, UK; 6 Neuroscience and Mental Health Institute and BRAIN unit Cardiff University, Cardiff, UK; 7 Huntington's Disease Centre, University College London, London, UK

**Keywords:** DEMENTIA, MOVEMENT DISORDERS, HUNTINGTON'S, NEUROGENETICS

## Abstract

**Background:**

Genetic testing for Huntington’s disease (HD) was initially usually positive but more recently the negative rate has increased: patients with negative HD tests are described as having HD phenocopy syndromes (HDPC). This study examines their clinical characteristics and investigates the genetic causes of HDPC.

**Methods:**

Clinical data from neurogenetics clinics and HDPC gene-panel data were analysed. Additionally, a subset of 50 patients with HDPC underwent whole-genome sequencing (WGS) analysed via Expansion Hunter and Ingenuity Variant Analysis.

**Results:**

HDPC prevalence was estimated at 2.3–2.9 per 100 000. No clinical discriminators between patients with HD and HDPC could be identified. In the gene-panel data, deleterious variants and potentially deleterious variants were over-represented in cases versus controls. WGS analysis identified one *ATXN1* expansion in a patient with HDPC.

**Conclusions:**

The HDPC phenotype is consistent with HD, but the genotype is distinct. Both established deleterious variants and novel potentially deleterious variants in genes related to neurodegeneration contribute to HDPC.

## Introduction

 Huntington’s disease (HD) is the most common inherited adult-onset neurodegenerative disorder with a prevalence of approximately 12.4/100 000 people, characterised by a combination of movement, cognitive and psychiatric symptoms^1^; the presentation may be heterogeneous. Historically, only 1% of patients received a negative genetic HD test,^2^ however, the negative test rate is thought to have increased. These patients are said to have HD phenocopy (HDPC) syndromes; studying them may provide valuable insights into the causes, networks and pathophysiology of HD and other neurodegenerative diseases. This study sought to clinically define HDPC syndromes and uncover potential genetic factors contributing to their development; we thereby hope to better understand HD and HDPC syndromes enabling the development of more effective diagnostic and treatment strategies for affected individuals.

## Methods

We analysed the clinical records of 151 patients in two neurogenetics clinics at Queen Square between 2016 and 2018, recording patients’ clinical symptoms at the time of genetic testing or symptoms reported at the onset of motor symptoms for pre-symptomatic HD testing. Results were analysed using SPSS V.26 (Fisher’s exact test) and R (logistical regression, conditional probabilities). To improve statistical comparability and applicability, 50 additional patients from the University College London genetic HDPC cohort who had received negative HD test results at Queen Square were included in the analysis.

Furthermore, we re-analysed a previously published dementia gene-panel applied to 552 patients with HDPC screening for deleterious variants in genes associated with dementia (*APP, CHMP2B, CSF1R, DNMT1, FUS, GRN, HTRA1, ITM2B, MAPT, NOTCH3, PRNP, PSEN1, PSEN2, TARDBP, TREM2, TYROBP* and *VCP*), with additional tests for *C9orf72* expansions and *PRNP* octapeptide repeats.^3^ Variants were classified following previously published criteria,^4^ which followed the guidelines published by the American College of Medical Genetics and Genomics and the Association for Molecular Pathology in 2015^3^, with some added clarifications specific to a dementia cohort. This classification used all available evidence to classify variants as benign, likely benign, variant of uncertain significance (VUS), likely pathogenic or pathogenic, and included an additional ‘potentially deleterious’ category of VUS found on gnomAD at <1 in 5000 and with at least some additional evidence of pathogenicity. We also performed whole-genome sequencing (WGS) on a subset of 50 patients chosen for their clinical similarity to HD (defined by the HDPC score, awarding a point each for the presence of a movement disorder, cognitive decline and psychiatric problems, respectively), the strength of their family history of neurological and/or psychiatric disease, their age at onset, whether neuropathological data was available and the number of years since they were last seen in clinic (for detailed selection criteria see online supplemental table S1). Family history was stratified using the Goldman score^5 6^ (GS), ranging from a strong autosomal family history (Goldman score 1) to no or unknown family history (Goldman score 4 and 4,5); no neuropathological data were available. Samples were sequenced at Edinburgh Genomics: Clinical Genomics using Illumina SeqLab and Genologics Clarity LIMS X Edition (read length 150 bp, average coverage 39.7×) and aligned to the Genome Reference Consortium Human Build 38. All samples were screened using ExpansionHunter ^7^ for known expansions and duplications in *AR, ATN1, ATXN1, ATXN2, ATXN3, ATXN7, ATXN10, C9ORF72, CACNA1A, CBL, CSTB, DMPK, DMPK, FMR1, FXN, HTT, JPH3 and PPP2R2B*. Samples without detected expansions were analysed using Ingenuity Variant Analysis software (IVA, www.qiagenbioinformatics.com, V.5.4.20190308, Ingenuity Systems). Variants found at a minimum frequency of 0.1% in healthy public genomes such as Gnomad^7^ were excluded from the analysis; only variants with high call quality, and predicted to be deleterious, likely deleterious, or of uncertain significance were included, as well as those predicted to cause either gain-of-function or loss-of-function of a gene. A number of subsequent filters were explored including variants linked to neurological disease, variants predicted to be deleterious by the IVA algorithm and variants linked to cell functioning in HD as per the IVA algorithm.

## Results

**Figure 1:**
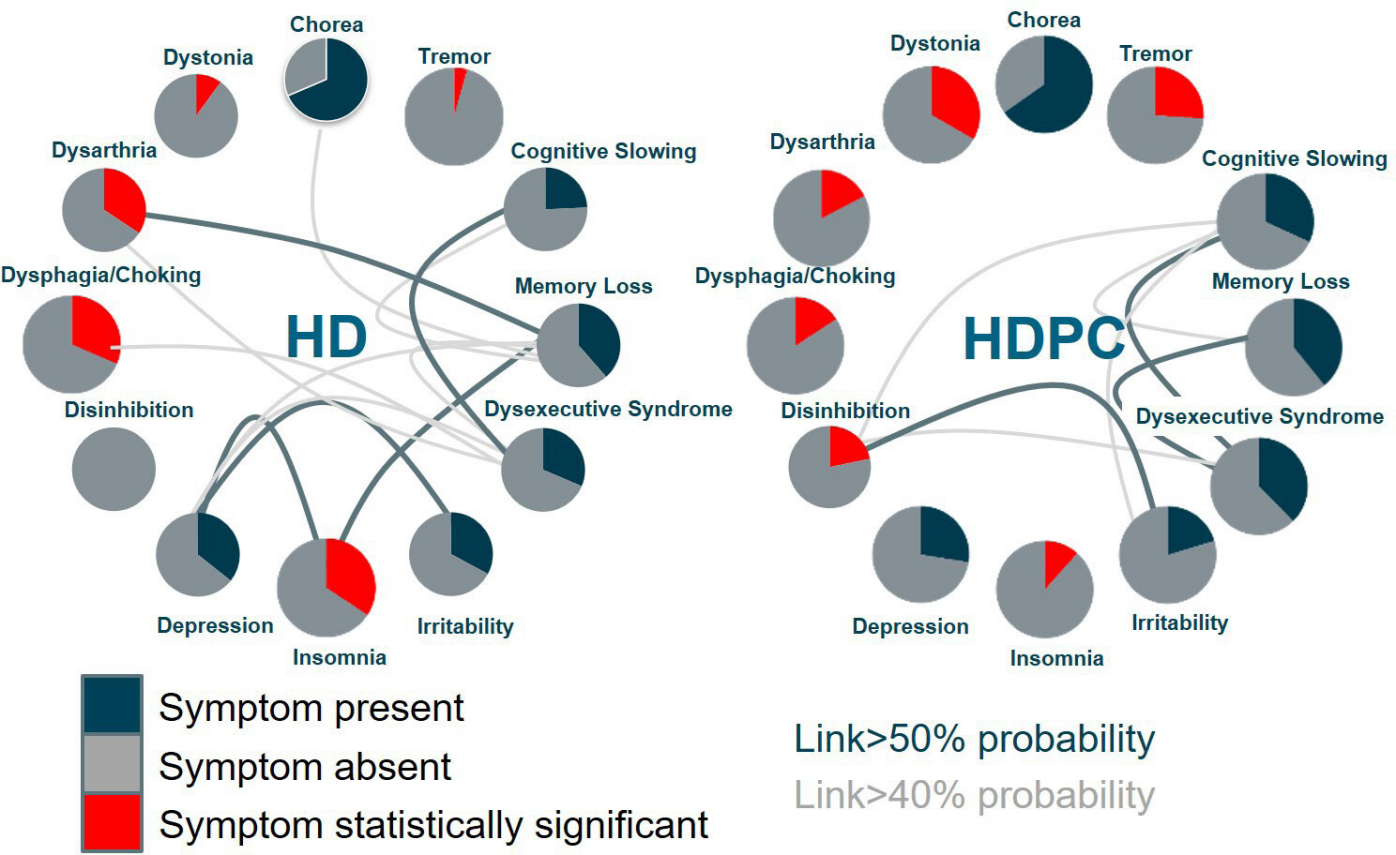
HD and HDPC display different patterns of concurrent symptoms: each symptom circle shows the percentage of patients in each group presenting with a given symptom; the circle is highlighted in red if the symptom differed significantly between the two groups (p≤0.05, logistical regression). Given the high number of included symptoms, none of the results withstood Bonferroni’s correction for multiple comparisons, but the results are interesting from an exploratory vantage point. The likelihood of a patient presenting with any given symptom pair was calculated using conditional probabilities, which express the likelihood that one symptom occurs given the presence of another in any given patient. If the likelihood is bilaterally equal or higher than 40%, a faint grey line connects the two symptoms, if it is equal or higher than 50%, the connecting line is emphasised in dark grey. All statistical analyses were done in R V.3.5.1 (https://www.rproject.org/) using the base package and custom written scripts. HD, Huntington’s disease; HDPC, HD phenocopy syndromes.

Out of 151 screened patients, 89 patients had sufficient data to be analysed. Out of these, 78.7% were found to carry the HTT expansion; among the patients with HDPC (21.3%), three were later diagnosed with Parkinson’s disease and spino-cerebellar ataxias (SCA 1 and SCA 17). The proportion of female HD and patients with HDPC was comparable, with 52.8% and 52.6%, respectively, and similar clinical presentations including chorea, depression and memory loss (see online supplemental table S2). On average, patients with HD experienced motor onset at a younger age and reported more dysphagia/choking, dysarthria and insomnia, while patients with HDPC showed a higher prevalence of tremor, dystonia and disinhibition. When considering symptom combinations, patients with HD more often exhibited combinations of depression and irritability, or early orolingual involvement with dysarthria, dysphagia or choking in patients with cognitive problems or hyperkinetic limb movements (see figure 1). Combined insomnia, memory loss and depression were also more common in HD than in patients with HDPC. Overall, patients with HD showed stronger connections between unrelated, diverse symptoms, while patients with HDPC demonstrated stronger connections between associated symptoms, such as disinhibition and irritability and executive dysfunction and memory loss. Rates of chorea were similar in both cohorts. Based on this patient series estimating the rate of HDPC syndromes at approximately 20% of suspected HD cases and given HD prevalence at 10.6–13.7/100 000,^8^ the prevalence of HDPC syndromes can be estimated to be approximately 2.5/100 000.^9^

Additionally, we re-analysed our published gene-panel dataset^4^ focusing on 552 patients with HDPC included in the DemMot cohort. 57.8% patients with HDPC were women, with an average age at onset (AAO) of 55.2 years. 4% had a GS of 1, 1.6% had a GS of 2, 2.9% had a GS of 3, 13.4% had a GS of 3.5, 37.5% had a GS of 4 and 40.6% had a GS of 4.5^3^. Clinical information was available for 361 patients, 77.1% of whom had a movement disorder, 37.4% had a cognitive decline and in 12.3% had psychiatric symptoms. Our reanalysis revealed deleterious and likely deleterious variants in 20 (3.62%) HDPC cases and 3 (0.66%) in controls (p=0.0016, χ^2^ test). Deleterious variants found in HDPC cases included 10 *C9orf72* expansions, *MAPT* Arg406Trp, *MAPT* Gly303Ser, *MAPT* c.915+19C>G, GRN Val411Alafs, *ITM2B* Ter267Argext*11, *PRNP* Tyr163Ter, *PSEN1* Ala434Thr, *PSEN1* Arg278Ile, *PSEN1* Ile437Val and *PSEN2* Val150Met (see table 1), including three novel but no concurrent variants. Of these, seven patients had a documented movement disorder, six had cognitive symptoms and three had psychiatric symptoms. Deleterious variants were least likely to be identified in patients with HDPC compared with other neurodegenerative diseases. In addition, 22 (4.0%) potentially deleterious variants were observed in HDPC cases versus (6 (1.31%) in controls (p=0.01730, χ^2^ test, see online supplemental table S3). While the available data is insufficient to classify these variants as disease-causing,^4^ they were found in excess in cases versus controls in the whole dataset (p=0.0039, OR: 3.2, 95% CI (1.39, 7.28)) and most likely to be identified in patients with HDPC (p=0.00012; OR: 4.63, 95% CI (1.92, 11.16)) compared with patients with Alzheimer's disease (p=0.0129; OR: 2.88, 95% CI (1.21, 6.89)) or frontotemporal dementia (p=0.0099; OR: 3.06, 95% CI (1.25, 7.47)).

**Table 1:** Summary of deleterious and likely deleterious variants identified in patients with HDPC in the gene-panel dataset. Shown are the variants and details of patients in whom they were identified, including sex, age at onset, the Goldman score as a measure of family history, as well as the HDPC+score (HDPC score plus positive family history) as a measure of how HD-like these patients were. Missing data is displayed as 666

Variant	Male	AAO	Goldman	Novel	HDPC+score
C9orf72	0	50	4.5	No	1
C9orf72	0	59	1	No	2
C9orf72	0	40	3	No	2
C9orf72	0	50	4.5	No	3
C9orf72	0	61	3	No	3
C9orf72	0	50	1	No	4
C9orf72	1	70	4	No	No information
C9orf72	0	56	4	No	No information
C9orf72	0	61	4.5	No	No information
C9orf72	1	25	4	No	No information
GRN Val411Alafs	0	63	4.5	No	0
ITM2B Ter267Argext*11	0	49	3	No	No information
MAPT c.915+19C>G	0	65	4.5	No	2
MAPT Arg406Trp	0	60	4	No	No information
MAPT Gly303Ser	0	666	4.5	Yes	No information
PRNP Tyr163Ter	0	52	4	No	2
PSEN1 Ala434Thr	1	43	4.5	Yes	2
PSEN1 Ile437Val	0	66	4.5	No	2
PSEN1p.Arg278Ile	0	28	3.5	0	1
PSEN2 Val150Met	1	30	4.5	1	1

AAO, age at onset; HD, Huntington’s disease; HDPC, HD phenocopy syndromes.

In the WGS dataset, 58% were women, with an average AAO of 51.2 years, a median GS of 3.5, and a mean HDPC plus positive family history score of 2.86/4. 92% of patients presented with a movement disorder (56% with chorea), 76% had cognitive decline, and 54% had psychiatric symptoms (see online supplemental table S4). The sample average coverage was 39.7×. Using ExpansionHunter,^10^ an Ataxin-1 expansion, known to cause SCA1, an established HDPC syndrome,^11 12^ was identified in one patient (see online supplemental figure S1), since clinically confirmed. The patient developed gait and coordination problems at 40 years and had a positive family history. No other expansions were found in the remaining samples. Analysis of the 49 remaining samples on the IVA platform found an average of 5 334 908 variants per sample (range 5 230 677–6 331 549). Only known deleterious and novel variants were considered for further analysis; no further deleterious variants in genes linked to neurodegeneration, dementia, chorea, other movement disorders or psychiatric disease could be identified.

## Discussion

The *HTT* expansion test was initially positive in 99% of tested patients,^2 11^ however, as HD testing became more accessible, the HD phenotype expanded and HD testing was applied to patients with atypical symptoms, leading to questions about whether testing for HD in these cases was appropriate.^13^ Here, we could establish that HD and HDPC remain clinically indistinguishable. The lack of any relevant family history of cognitive, psychiatric or motor symptoms currently appears to most strongly suggest an HDPC syndrome. Once negative results are obtained for *HTT*, *C9orf72* and *TBP* expansions, the diagnostic rate in patients with HDPC plummets.^4 14 15^ Our data suggests that novel, unusual variants in dementia genes could confer atypical features, expand the phenotypic spectrum of dementias, and further elucidate the mechanisms at work in subcortical dementias.

The two-tiered approach to genetic investigations in this study offers lessons for regular clinical practice. The diagnostic rate of 20/552 (3.62%) in our dementia-gene panel HDPC cohort and 1/50 (2.0%) in our WGS HDPC cohort was much lower than for other cohorts in our dementia-gene panel study (eg, 6.8% in Alzheimer’s disease and 20.3% in frontotemporal dementia)^4^ and much lower than the general diagnostic rates for clinical WGS, previously estimated at 34%.^9^ This may be due to the causes of HDPC syndromes. A classical dementia gene-panel may only allow the identification of a few established deleterious variants in genes known to be linked to dementia, while novel and understudied variants may play an outsize role in HDPC syndromes. Genes not previously established to cause neurodegenerative disease, structural, and non-coding variants, may also be involved. Unless WGS analysis allows for the exploration of atypical causes of dementia, yields may remain low, even when computationally screening WGS data for expansions and the rate of variants of uncertain significance may be significant. Bioinformatics analysis methods, such as ExpansionHunter, are one of the most accessible forms of screening WGS data for expansion disorders while also searching for single nucleotide polymorphisms (SNPs) and smaller indels^10^ with high sensitivity and specificity^16^ and are not restricted to PCR-free WGS datasets. Careful genetic counselling is however key, taking into account patients’ mental capacity and implications for the patient’s relatives.^17^ A genetic diagnosis may assist in the management not only of the patient themselves but also of at-risk family members, especially given the increasing emergence of clinical trials for genetically-defined neurodegenerative disease and pre-implantation diagnostics.

Study limitations include the retrospective analysis of potentially flawed clinical documentation, and an analysis heavily focused on exomes of genes already linked to neurodegeneration, leaving scope for further analyses. In summary, the HDPC phenotype is consistent with HD, but the genotype is distinct. Further research is needed to explore the role of genes not previously associated with dementia and to examine unexplored data.

## Supplementary material

10.1136/jnnp-2024-333602 online supplemental file 1

## References

[1] Bates GP, Dorsey R, Gusella JF (2015). Huntington disease. Nat Rev Dis Primers.

[2] Andrew SE, Goldberg YP, Kremer B (1994). Huntington disease without CAG expansion: phenocopies or errors in assignment?. Am J Hum Genet.

[3] Directors A (2015). ACMG policy statement: updated recommendations regarding analysis and reporting of secondary findings in clinical genome-scale sequencing. Genet Med.

[4] Koriath C, Kenny J, Adamson G (2020). Predictors for a dementia gene mutation based on gene-panel next-generation sequencing of a large dementia referral series. Mol Psychiatry.

[5] Goldman JS, Farmer JM, Wood EM (2005). Comparison of family histories in FTLD subtypes and related tauopathies. Neurol (ECronicon).

[6] Rohrer JD, Guerreiro R, Vandrovcova J (2009). The heritability and genetics of frontotemporal lobar degeneration. Neurol (ECronicon).

[7] Karczewski KJ, Francioli LC, Tiao G (2020). The mutational constraint spectrum quantified from variation in 141,456 humans. Nat New Biol.

[8] McColgan P, Tabrizi SJ (2018). Huntington’s disease: a clinical review. Eur J Neurol.

[9] Ewans LJ, Minoche AE, Schofield D (2022). Whole exome and genome sequencing in mendelian disorders: a diagnostic and health economic analysis. Eur J Hum Genet.

[10] Dolzhenko E, van Vugt JJFA, Shaw RJ (2017). Detection of long repeat expansions from PCR-free whole-genome sequence data. Genome Res.

[11] Wild EJ, Mudanohwo EE, Sweeney MG (2008). Huntington’s disease phenocopies are clinically and genetically heterogeneous. Mov Disord.

[12] Schneider SA, Bird T (2016). Huntington’s Disease, Huntington’s Disease Look-Alikes‎, and Benign Hereditary Chorea: what’s New?. Mov Disord Clin Pract.

[13] Feigin A, Talbot K (2014). Expanding the genetics of huntingtonism. Neurol (ECronicon).

[14] Sułek-Piatkowska A, Krysa W, Zdzienicka E (2008). Searching for mutation in the JPH3, ATN1 and TBP genes in Polish patients suspected of Huntington’s disease and without mutation in the IT15 gene. Neurol Neurochir Pol.

[15] Keckarević M, Savić D, Svetel M (2005). Yugoslav HD phenocopies analyzed on the presence of mutations in PrP, ferritin, and Jp-3 genes. Int J Neurosci.

[16] Ibañez K, Polke J, Hagelstrom RT (2022). Whole genome sequencing for the diagnosis of neurological repeat expansion disorders in the UK: a retrospective diagnostic accuracy and prospective clinical validation study. Lancet Neurol.

[17] Koriath CAM, Kenny J, Ryan NS (2021). Genetic testing in dementia - utility and clinical strategies. Nat Rev Neurol.

