# Supplementary material for: Huntington’s disease phenocopy syndromes revisited: a clinical comparison and next-generation sequencing exploration
Source: J Neurol Neurosurg Psychiatry. 2024 Oct 23;96(5):e333602. doi: 10.1136/jnnp-2024-333602 (PMC12015005; doi:10.1136/jnnp-2024-333602)

Supplementary Data

Table S1: Ranking and weighting factors for the selection of HD phenocopy samples for whole-genome sequencing

Each category of the criteria for each sample is awarded a number of points; these are then multiplied by the respective criterion's weighting factor and added up to generate a total score for each sample. The maximum achievable score in this ranking is 100 points. No neuropathological data was available for any of the 50 patients chosen for WGS.

Criteria	Category	Points awarded
**HD phenocopy score**	3	30
	2	20
	1	5
**Goldman score**	1	20
	2	10
	3	4
	3.5	2
	4	0
**Age at onset**	≤35	30
	≤45	20
	≤55	10
	≤65	5
**Neuropathology available?**	Yes	15
	No	0
**Years since seen in clinic**	Last 2 years	5
	Last 3 years	2
	Last 5 years	1
**Maximum Total**		100

Table S2: Characteristics of HD/HDPC patients in the clinical comparison cohort

151 patients from two Neurogenetics clinics run by the same consultant were analysed. Out of these, 62 patients were excluded for insufficient data, including not having had an HD test. Out of the remaining 89 patients 78.7% were found to carry the HTT expansion, while among the HDPC patients (21.3%), three were later diagnosed with Parkinson's disease and spino-cerebellar ataxias (SCA 1 and SCA 17).

Symptom percentages are based on their being listed in the clinical notes and letters. Where possible a separate AAO for motor, cognitive and psychiatric symptoms was established from the notes (AAO Mot, AAO Cog, AAO Psy, respect.). In addition, the HDPC score was calculated for each patient based on whether they were displaying any symptoms in the cognitive, psychiatric and motor domains (cognitive, psychiatric, and motor HDPC score, respectively); this was used as a measure of how HD-like patients' clinical presentations were in terms of affecting different functional domains

A) **Characteristics of the cohort**

Characteristic or Symptom	HD (%HD)	HDPC (%HDPC)	p-value (uncorrected Chi-square/t-test)
Numbers	70	69	
Male	33 (47.1%)	29 (42.0%)	n.s.
GS	2 (median)	4 (median)	n.s.
AAO Mot	44.9 years	50.4 years	n.s.
AAO Cog	40.0 years	51.1 years	n.s.
AAO Psy	37.4 years	47.0 years	n.s.
Total HDPC + FH score	2.9	2.6	n.s.
Psychiatric HDPC score	0.7	0.6	n.s.
Motor HDPC score	0.9	0.9	n.s.
Total HDPC score (noFHx)	2.1	2.2	n.s.

B) **Symptoms suggestive of HD**

Characteristic or Symptom	HD (%HD)	HDPC (%HDPC)	p-value (uncorrected Chi-square/t-test)
Insomnia	24 (34.3%)	8 (11.6%)	0.002
Dysphagia /Choking	22 (31.4%)	8 (11.6%)	0.007
Dysarthria	24 (34.3%)	12 (17.4%)	0.033
Falls	23 (32.9%)	14 (20.3%)	n.s.
Irritability	23 (32.9%)	14 (20.3%)	n.s.
Depression	25 (35.7%)	19 (27.5%)	n.s.
Ocular Saccades	22 (31.4%)	16 (23.2%)	n.s.
Agitation	6 (8.6%)	3 (4.3%)	n.s.
Apathy	7 (10%)	4 (5.8%)	n.s.
Weight Loss	8 (11.4%)	5 (7.2%)	n.s.
Rigidity	11 (15.7%)	8 (11.6%)	n.s.
Anxiety	18 (25.7%)	15 (21.7%)	n.s.
Ocular Smooth Pursuit	17 (24.3%)	14 (20.3%)	n.s.
Gait abnormality	38 (54.3%)	35 (50.7%)	n.s.
Chorea	48 (68.6%)	45 (65.2%)	n.s.

Characteristic or Symptom	HD (%HD)	HDPC (%HDPC)	p-value (uncorrected Chi-square/t-test)
**Hypersomnia**	1 (1.4%)	0 (0%)	n.s.
**Bradykinesia**	12 (17.1%)	11 (15.9%)	n.s.

C) Symptoms less suggestive of HD

Characteristic or Symptom	HD (%HD)	HDPC (%HDPC)	p-value (uncorrected Chi-square/t-test)
**Dystonia**	7 (10%)	23 (33.3%)	0.001
**Tremor**	3 (4.3%)	18 (26.1%)	0.00031
**Disinhibition**	0 (0%)	15 (21.7%)	0.000012
**Cognitive HDPC score**	0.5	0.6	n.s.
**Cognitive Slowing**	17 (24.3%)	22 (31.9%)	n.s.
**Dysexecutive Syndrome**	22 (31.4%)	26 (37.7%)	n.s.
**Limb weakness**	0 (0%)	4 (5.8%)	n.s.
**Spasticity**	0 (0%)	4 (5.8%)	n.s.
**Supranuclear Palsy**	1 (1.4%)	5 (7.2%)	n.s.
**Myoclonus**	3 (4.3%)	6 (8.7%)	n.s.
**OcularRangeofMovemen**	3 (4.3%)	6 (8.7%)	n.s.
**Paranoia**	1 (1.4%)	4 (5.8%)	n.s.
**Loss of Empathy**	0 (0%)	3 (4.3%)	n.s.
**Sensory Neuropathy**	0 (0%)	3 (4.3%)	n.s.
**Pain**	0 (0%)	3 (4.3%)	n.s.
**Sweet Tooth/Change in dietary habits**	0 (0%)	3 (4.3%)	n.s.
**Obsessive Behaviour**	3 (4.3%)	5 (7.2%)	n.s.
**Disorientation/Navigatio**	2 (2.9%)	4 (5.8%)	n.s.
**Hallucinations**	1 (1.4%)	3 (4.3%)	n.s.
**Ataxia**	12 (17.1%)	13 (18.8%)	n.s.
**Asymmetry**	1 (1.4%)	2 (2.9%)	n.s.
**Babinski**	1 (1.4%)	2 (2.9%)	n.s.
**Delusions**	2 (2.9%)	3 (4.3%)	n.s.
**Bladder/Bowel Dysfunction**	0 (0%)	1 (1.4%)	n.s.
**Cortical Blindness**	0 (0%)	1 (1.4%)	n.s.
**Nystagmus**	0 (0%)	1 (1.4%)	n.s.
**Memory Loss**	27 (38.6%)	27 (39.1%)	1
**Lost Reflexes**	0 (0%)	0 (0%)	N/A - constant
**Motor Neuropathy**	0 (0%)	0 (0%)	N/A - constant

Table S3: Potentially deleterious variants detected in the HDPC cohort

In the gene panel data, 21 variants were classified as “potentially deleterious”, meaning they do not fulfil the modified ACMG criteria to be classified as deleterious or likely deleterious, but that there is some evidence that they may be pathogenic. This may be related to being located in a binding domain, a major amino acid change or similar. AAO = age at onset. Goldman score = measure of heritability. HDPC score = one point each for cognitive, psychiatric or motor symptoms, as well as evidence of a positive family history.

Variant	Male	AAO	Goldman	HDPC+ score
**CSF1R Arg549Cys**	Female	71	4	N/A
**FUS Gly227_Gly229del**	Female	71	4	N/A
**FUS Gly229Ser**	Female	71	4	1
**FUS Pro431Leu**	Male	N/A	4.5	N/A
**GRN Arg478His**	Female	69	1	N/A
**GRN Asp33Glu**	Male	70	3.5	3
**GRN Gly148Arg**	Male	59	3,5	N/A
**GRN Pro166Leu**	Female	73	4	N/A
**NOTCH3 Asp1869Gly**	N/A	N/A	666	N/A
**PSEN1 His46Tyr**	Female	64	3,5	3
**PSEN1 Ile148Val**	Female	19	4	1
**PSEN2 Asp431Glu**	Male	25	4,5	2
**SERPINI1 Leu307Ser**	Male	40	4	N/A
**SERPINI1 Ser142Gly**	Female	58	4,5	1
**SERPINI1 Ser142Gly**	Female	58	4,5	1
**SERPINI1 Val288Ile**	Male	0	4,5	N/A
**SQSTM1 Thr339Ile**	Female	40	4,5	1
**SQSTM1 Val240Ala**	Female	55	1	4
**SQSTM1 Val271Ile**	N/A	N/A	N/A	N/A
**VCP c.1359+8C>T**	Female	73	4,5	2
**VCP c.-254_-253insCGCTGCCGCTGCCGCTGC**	Male	50	4,5	N/A
**VCP 5'UTR c.-221_-216delGCTGCC**	Male	40	1	2

Table S4: Details of patient samples selected for whole-genome sequencing

Based on the selection criteria described in Table 1, 50 samples were selected for whole-genome sequencing; their details are described in this table. Sex, age at onset (AAO) and the strength of the family history (Goldman score, see Figure II-1) were noted, and the clinical notes scoured for symptoms the patients developed in their lifetime, as well as the last time they presented in clinic at the National Hospital for Neurology and Neurosurgery (NHNN). Based on this information and their respective score, patient samples were shortlisted and selected. HDPS Movement + HDPS Movement Disorder; HDPS Cognitive = HDPS Cognitive Decline; HDPS Psychiatric = HDPS Psychiatric Disturbance; Neuropath = Neuropathology available

HDPC Sample	Sex	AAO	Goldman Score	HDPS Movement	HDPS Cognitive	HDPS Psychiatric	HDPS Total	Neuro-path	Chorea present	Last Clinic (years)	Total score
**1**	Fe	43	1	1	1	1	3	no	yes	2	85
**2**	Fe	55	1	1	1	1	3	no	yes	2	85
**3**	Mal	59	1	1	1	1	3	no	no	5	81
**4**	Mal	61	1	1	1	0	2	yes	no	2	80
**5**	Fe	69	2	1	1	1	3	no	yes	2	75
**6**	Fe	45	2	1	1	1	3	no	yes	2	75
**7**	Mal	50	3.5	1	1	1	3	no	yes	2	67
**8**	Fe	49	3.5	1	1	1	3	no	yes	2	67
**9**	Mal	59	4	1	1	1	3	no	yes	2	65
**10**	Mal	53	4	1	1	1	3	no	no	2	65
**11**	Mal	70	4	1	1	1	3	no	yes	2	65
**12**	Fe	39	1	1	1	0	2	no	yes	2	65
**13**	Fe	49	4.5	1	1	1	3	no	no	2	65
**14**	Mal	43	4	1	1	1	3	no	yes	2	65
**15**	Mal	64	4	1	1	1	3	no	yes	2	65
**16**	Fe	11	1	1	0	1	2	no	yes	2	65
**17**	Mal	43	3.5	1	1	1	3	no	no	3	64
**18**	Mal	53	3.5	1	1	1	3	no	no	53	63
**19**	Mal	39	3.5	1	1	1	3	no	no	-	62
**20**	Mal	47	4	1	1	1	3	no	no	3	62
**21**	Fe	43	1	0	1	1	2	no	no	-	60
**22**	Fe	69	4	1	1	1	3	no	yes	-	60
**23**	Mal	33	4	1	1	1	3	no	yes	-	60
**24**	Fe	34	2	1	0	1	2	no	no	2	55
**25**	Fe	55	2	1	1	0	2	no	no	2	55
**26**	Mal	75	3.5	1	1	0	2	no	yes	2	47
**27**	Fe	63	3.5	1	1	0	2	no	yes	2	47
**28**	Fe	66	3.5	1	0	1	2	no	yes	2	47

HDPC Sample	Sex	AAO	Goldman Score	HDPS Movement	HDPS Cognitive	HDPS Psychiatric	HDPS Total	Neuro-path	Chorea present	Last Clinic (years)	Total score
**29**	Mal	44	3.5	1	1	0	2	no	no	2	47
**30**	Fe	62	3.5	1	1	0	2	no	no	2	47
**31**	Fe	34	3.5	1	0	1	2	no	yes	2	47
**32**	Fe	70	3.5	1	0	1	2	no	yes	2	47
**33**	Fe	65	1	1	1	0	2	no	no	5	46
**34**	Mal	16	4	0	1	1	2	no	no	2	45
**35**	Fe	68	4	1	0	1	2	no	yes	2	45
**36**	Fe	46	3	1	1	0	2	no	no	5	45
**37**	Mal	77	4	1	1	0	2	no	yes	2	45
**38**	Mal	51	4	1	1	0	2	no	yes	2	45
**39**	Mal	64	4	1	1	0	2	no	yes	2	45
**40**	Fe	53	4	1	1	0	2	no	yes	2	45
**41**	Fe	46	4	1	1	0	2	no	no	2	45
**42**	Fe	53	4	1	1	0	2	no	yes	2	45
**43**	Fe	58	4	1	1	0	2	no	no	2	45
**44**	Fe	22	1	1	0	0	1	no	yes	2	35
**45**	Fe	52	1	1	0	0	1	no	yes	2	35
**46**	Fe	52	1	1	0	0	1	no	yes	2	35
**47**	Mal	40	1	1	0	0	1	no	no	2	35
**48**	Mal	67	1	0	1	0	1	no	no	2	35
**49**	Fe	35	1	1	0	0	1	no	no	-	30
**50**	Fe	45	1	0	0	0	0	0	no	5	21

Figure S1: ExpansionHunter® identifies a repeat expansion in the *ATXN1* gene

ExpansionHunter® correctly identified an expansion in the *ATXN1* gene, as can be observed on this output from the programme. All but one sample were estimated to have repeat sizes well below the 39 CAG repeats found in patients with spino-cerebellar ataxia type 1 (SCA1).

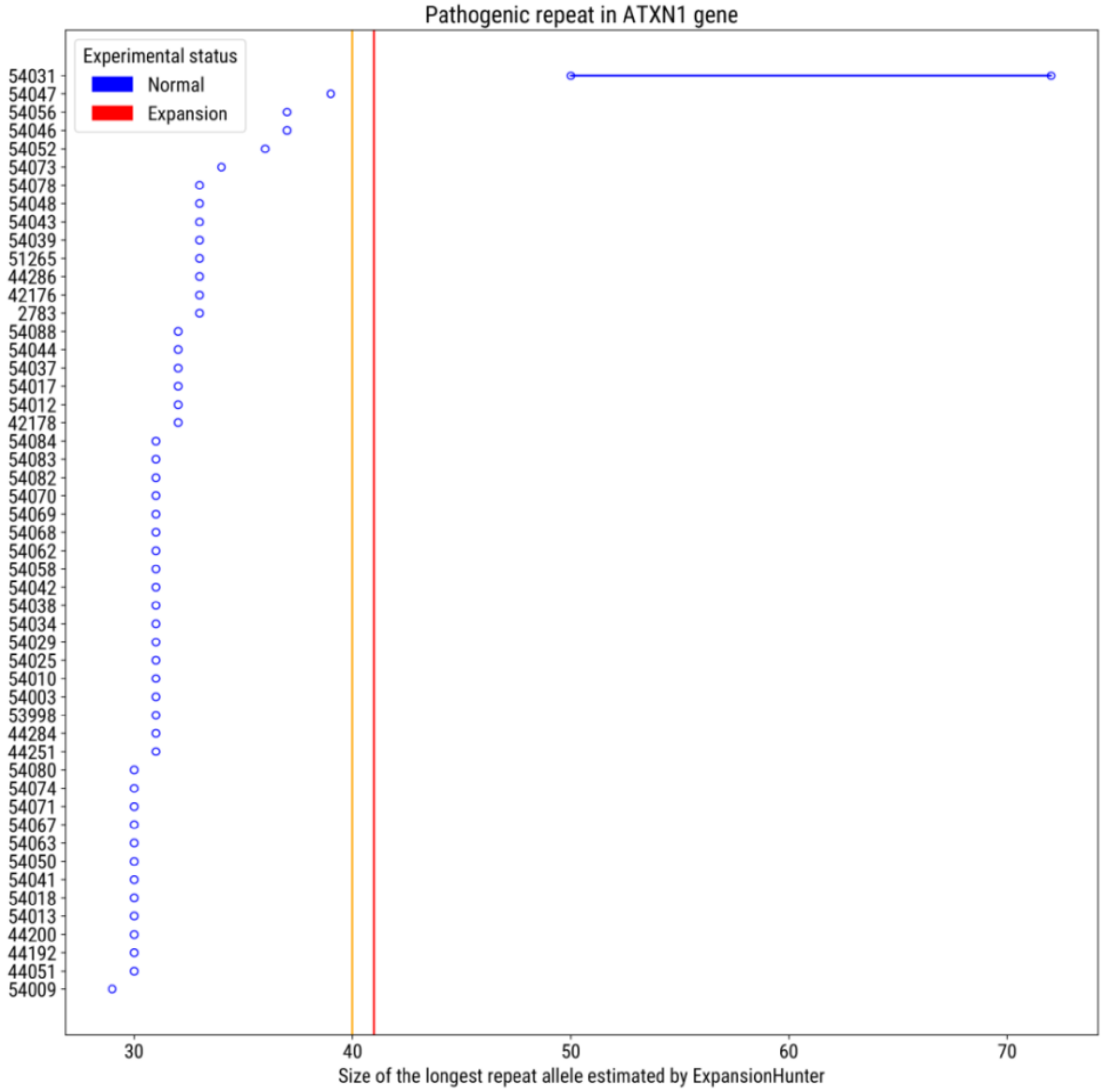

Supplement: online supplemental file 1 [file jnnp-96-5-s001.pdf]
